# When they talk about *motherhood*: a qualitative study of three groups’ perceptions in a Swedish child health service context

**DOI:** 10.1186/s12939-016-0387-8

**Published:** 2016-06-24

**Authors:** Erik Masao Eriksson, Kristin Eliasson, Andreas Hellström, Sylvia Määttä, Lisa Vaughn

**Affiliations:** Department of System Development and Strategy, Western Region of Sweden/Västra Götalandsregionen, Regionens Hus, SE-405 44 Göteborg, Sweden; Centre for Healthcare Improvement and Division of Service Management and Logistics, Chalmers University of Technology, Teknikens ekonomi och organisation, SE-412 96 Göteborg, Sweden; Cincinnati Children’s Hospital Medical Center/University of Cincinnati College of Medicine, 3333 Burnet Ave ML 2008, Cincinnati, OH 45229 USA

**Keywords:** Motherhood, Child health, Social construction, User perceptions, Country of birth, Sweden

## Abstract

**Background:**

In light of the growing emphasis on individualization in healthcare, it is vital to take the diversity of inhabitants and users into consideration. Thus, identifying shared perceptions among group members may be important in improving healthcare that is relevant to the particular group, but also perceptions of the staff with whom interactions take place. This study investigates how motherhood is perceived among three groups: Somali-born mothers; Swedish-born mothers; and nurses at Swedish child health centers. Inequities in terms of access and satisfaction have previously been identified at the health centers.

**Methods:**

Participants in all three groups were asked to finalize two statements about motherhood; one statement about perfect motherhood, another about everyday motherhood. The responses were analyzed using qualitative coding and categorization to identify differences and similarities among the three groups.

**Results:**

The responses to both statements by the three groups included divergences as well as convergences. Overall, biological aspects of motherhood were absent, and respondents focused almost exclusively on social matters. Working life was embedded in motherhood, but only for the Somali-born mothers. The three groups put emphasis on different aspects of motherhood: Somali-born mothers on the community; the Swedish-born mothers on the child; and the nurses on the mother herself. The nurses – and to some extent the Swedish-born mothers – expected the mother to ask for help with the children when needed. However, the Somali-born mothers responded that the mother should be independent, not asking for such help. Nurses, more than both groups of mothers, largely described everyday motherhood in positively charged words or phrases.

**Conclusion:**

The findings of this paper suggest that convergences and divergences in perceptions of motherhood among three groups may be important in equitable access and utilization of healthcare. Individualized healthcare requires nuance and should avoid normative or stereotypical encounters by recognizing social context and needs that are relevant to specific groups of the population.

## Background

In recent years, increased attention has been focused on concepts supporting the pivotal role of the *individual’s* perceptions in healthcare [1–3]. This focus has been accompanied and reinforced by the marketization of Western society and the prominence given to concepts of service management [4, 5]. In the 1980s, experts in the field of service quality argued for *quality* as a subjective perception, which varies from customer to customer [6]. The next decade saw an increased focus on the concept of *value* [7] as an individualized and even unique perception of the customer [8, 9].

Individualization in healthcare is often proclaimed in order to enhance the healthcare user’s position by becoming more participative and well-informed [10, 11], ranging from co-developing treatment plans to choosing healthcare providers [12, 13]. For multiple reasons, not all patients or inhabitants have the possibilities or prerequisites to be participative or well-informed [14, 15]. Barriers may be constituted due to language skills [16, 17], one’s economic situation [18], or long travelling distances [19]. Possibilities to be participative or well-informed may also be constrained by the provider’s normative or stereotypical expectations and perceptions [18]. For instance, Hedegaard et al. [10] found physicians to unconsciously be more amenable toward native Swedish-speaking than non-native speaking patients despite the latter group communicating more in align with “patient-centered care” (e.g. being well-informed, and actively asking questions). Stereotypes have also been reported in the setting of the current paper, the Swedish child health centers, in which “family” was heteronormatively assumed to consist of child, mother and father in information given to parents [18].

Given the above, Saha et al. [20] argued that individualization in healthcare must take the diversity of patients’ perspectives into consideration. Thus, identifying group members shared perceptions may be a first step in improving healthcare that is relevant to the particular group and grounded within their social context [16, 17]. The social context also implies that societal structures and norms influence human interaction [21, 22], thus it is also important to inquire about healthcare providers’ perceptions [23].

In the decentralized Swedish healthcare system, the national government is responsible for overall objectives and regulation. At the two local levels of government – county councils and municipalities – it is decided how healthcare is to be delivered given the local conditions [24]. Generally, the county councils are responsible for providing high quality healthcare through hospitals, primary care – including child health services – and dental care, whereas the municipalities are responsible for care for the elderly, people with physical disabilities or psychological disorders as well as school health [25]. Funding comes mainly from county council and municipal taxes, but also from out-of-pocket fees or national government grants [26]. The purpose of Swedish child health services is to promote children’s health, development, and well-being [27]. A local child health center offers voluntary child health promotion programs and free services for all preschool children (newborn to age 6 years) and their parent(s). The responsibility for carrying out the programs rests mainly with the nurse at the center, often a pediatric or district nurse. The nurse’s importance as a resource and support for parents has been recognized [28]. The needs of each family determine the frequency of appointments. Typically, there are 10 to 20 health appointments during the child’s first year, and then annual appointments until school-based healthcare providers take over these responsibilities [29]. Besides the care and assessment provided by the child health nurse, each center offers additional services, including vaccination programs, language tests, eye examinations, and parental education given in groups. In addition to the nurse, physicians and psychologists are seen regularly or when required. Despite this seeming standardization, there is an increasing recognition of the variation in services provided by the country’s child health centers [30].

Inequities have been identified in terms of access to child health services, for example difficulties in attracting fathers [31, 32] and unemployed mothers [33] to visit the centers. Other researchers have found that the centers do reach various groups, but do not always adequately meet the diverse support needs of different groups [34]. Inequities are also manifested as less satisfaction with provided services among mothers of low socio-economic status [35], and same-sex parents experiencing heteronormative communication [36]. In an attempt to coordinate services in the decentralized Swedish healthcare system, a government agency published national guidelines in 2014 [27], which emphasized the importance of including and addressing the needs of *all* parents. Subsequently, efforts have been made to change approaches and attitudes at the local centers [29]. For instance, in addressing the growing number of parents from Somalia, the nurses at one center worked with reflexivity of their own preunderstandings, resulting in better encounters with all families [23].

Research on motherhood in Sweden in the 2000s has often addressed gender equality and both parents’ responsibilities of parenting as well as opportunities to do paid work [37, 38] – independent of the mother’s country of birth [39] or specifically focused on Swedish-born mothers [40]. For the latter group, research has found that becoming a mother is more of an individualized life project as compared to mothers with Turkish background living in Sweden to whom becoming a mother was more of a collective project [41].

It has been reported that Sweden’s local child health centers often fail to attract immigrant mothers [33]. Sweden’s population of nearly 10 million people includes a substantial number (15%) born outside its borders [42]. Of all immigrants to Sweden in 2013, almost 10 % held Somali citizenship; the only groups that were larger were Syrian immigrants and returning Swedish citizens [43]. For two decades, Somalia has been one of the most common countries of origin for immigrants to Sweden [44]. However, according to an official report, Somalis as a group have had particular difficulty integrating into Swedish society [45]. Somalis living in Sweden have among the lowest employment rates [45, 46] and educational levels [46] of all groups. Findings of previous studies of Somali immigrants in Swedish healthcare include: dissatisfaction with healthcare encounters [47]; dissatisfaction with childbirth experiences [48, 49]; a relatively high proportion of Somali children with autism [50]; and a relatively high risk of vitamin D deficiencies [51]. Overall, Somalis’ perceptions of Swedish healthcare have not been well addressed [52].

The current manuscript is directed at examining the perceptions of motherhood as expressed by two groups of mothers – Swedish-born and Somali-born, and exploring the implications for individualized healthcare. This goal includes the recognition that those belonging to a particular group may share perceptions, and that healthcare nurses (the third group) also hold their own perceptions about mothers yet must recognize differing perceptions among various groups.

## Methods

### Setting and participants

This study took place in Western Sweden, in the country’s second largest region, which has over 1.6 million inhabitants [53]. To enhance understanding of different perceptions among groups – and not only among individuals – we asked 20 Somali-born mothers, 50 Swedish-born mothers, and 35 child health nurses to complete two statements about motherhood. The responses of the Swedish-born mothers and nurses were collected at two child health centers in two medium-sized cities in Western Sweden. Responses of the Somali-born mothers were initially collected at one child health center in a medium-sized city. In order to reach saturation [54], additional responses were collected at a local meeting place for parents located in a multicultural area of the region’s largest city. The diversity of settings for data collection was thought to contribute to a more robust level of saturation within each group of respondents.

The collected, de-identified demographic data revealed that all but two of 50 responding Swedish-born mothers had a partner, and they each had between one and nine children. Among the Somali-born mothers, eight of the 20 respondents had a partner, and they each had between one and seven children. The Swedish-born mothers were 22 to 40 years of age, and the Somali-born mothers were 20 to 34 years old.

### Data collection and analysis

Mothers and nurses were informed about the study both verbally and in writing. Verbal and written consent were obtained prior to data collection. All participants were informed about the study’s purpose; that participation was voluntary; and that they could withdraw anytime without consequence. Moreover, they were informed that all published information would be anonymous [54]. Using a similar data collection procedure as in previous research [55, 56], the participants were asked to complete two statements: 1) “To me, being a perfect mother means…”; and 2) “In everyday life, being a mother means…” The statements were chosen to identify disparities given the mothers’ life situations, and to compare their ideal conceptions of motherhood with what they believed was realistic. Each respondent could provide multiple responses to each statement, as shown in Table 1.

**Table 1:** Participants and Responses

Participants	Number of participants	Statement 1	Statement 2
		Number of responses	Number of responses
Child health nurses	35	125	101
Somali-born mothers	20	58	38
Swedish-born mothers	50	121	100
TOTAL	105	304	239

Analysis was inspired by the qualitative content analysis procedure of Graneheim and Lundman [57]. Analysis can be done on content close to the text (*manifest content*), or on the underlying meaning (*latent content*). In this study, the manifest content was analyzed. All responses (*n* = 543) were read through several times and coded by the research members EE and KE. The codes were clustered into two types of categories: general categories and group-specific categories (Swedish-born mothers, Somali-born mothers, child health nurses). Of particular interest were the emerging similarities and differences among the three groups. The constructed categories were discussed with the other research team members and further adjusted.

## Results

**Fig. 1:**
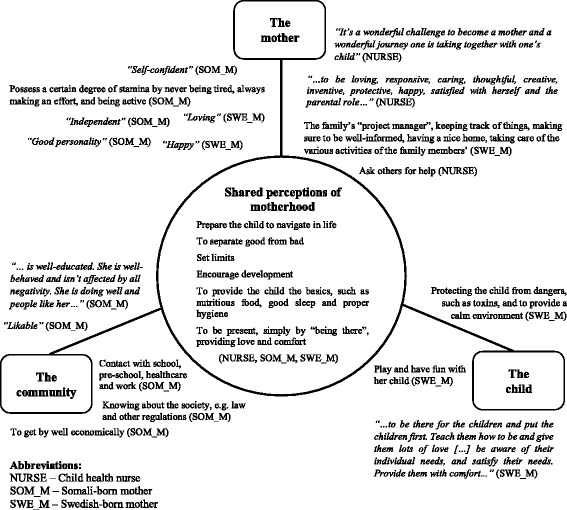
Focus of motherhood perceptions

Few differences were revealed between the responses to the statements of everyday and perfect motherhood. Consequently, the perceptions organized in Fig. 1 address responses among the groups that are common to both perfect and everyday motherhood. Perceptions of motherhood commonly expressed and shared among the three groups are presented in the circle in the middle. The three surrounding squares represent three distinct foci of motherhood as derived from the responses: 1) the mother; 2) the community; and 3) the child. The perceptions and quotations are distributed closest to the focus of motherhood mainly addressed. The respondents’ group categorization is indicated within brackets: child health nurses, Somali-born mothers, and Swedish-born mothers.

For all three groups, a mother was perceived as someone providing the basics, guiding the child, and explaining things, as exemplified in the mid-circle. Here, motherhood was expressed in terms of “Guiding the child in life – set limits” (nurse); or to make sure “[t]hat the children’s most basic needs are met, such as cooking, going to the toilet, and to rest” (Swedish-born mother); or to “love the child” (Somali-born mother). These and similar responses were common among all three groups, irrespective of addressing perfect or everyday motherhood.

Despite these similarities, there were also group-specific responses to motherhood. The nurses typically thought of a mother as someone capable of asking others for help. For instance, by “admit[ting] to her surroundings when it’s tough and hard work…”, or to “understand when she needs to ask for help.” Often “the partner” – or more rarely, “the father” – or parents-in-law were mentioned as providing this help. Moreover, the nurses often focused on motherhood in relation to the characteristics of mothers’ themselves, predominately by using positively charged words, such as “being loving*”*, or to be “caring, creative, inventive, friendly.”

Swedish-born mothers also used “positive” terms to describe the mother’s characteristics. Some Swedish-born mothers responded that a perfect mother “felt good.” Feeling good was expressed in relation to the child; a perfect mother should take good care of herself so she can take care of the child rather than for her own sake. Unlike the nurses’ focus on the mothers, the Swedish-born mothers talked about motherhood almost exclusively from the child’s perspective: “mak[ing] sure that one’s child is feeling okay”, or “… that she develops in every way and that she becomes a safe individual with good self-esteem and self-confidence.”

Somali-born mothers typically responded that a mother possessed a certain degree of stamina and was supposed to be active, “never get tired”, and always “make an effort.” Different from both the nurses’ and the Swedish-born mothers’ perceptions, the Somali-born mothers typically talked about motherhood as embedded in a community context, as mothers should have “good contact with school, teachers, school nurse, and physicians”.

To a great extent, the responses for perfect and everyday motherhood overlapped. However, a few differences were identified. The nurses used “positive” terms to describe the characteristics of both perfect and everyday motherhood. Swedish-born mothers were more nuanced when describing everyday motherhood, often using both negative and positive wording in tandem: “Often headache. Often feeling insufficient when the children are screaming. But then there is a glimmer and emotions of joy takes over, the pride and the happiness.”

More than the other groups, Somali-born mothers emphasized negative aspects of everyday motherhood more than they did for perfect motherhood. Here, motherhood was associated with having a “bad character,” “lying,” “being angry,” “lazy,” “tired,” “absent,” “impatient,” or “not taking care of herself.” Furthermore, she was *not* well-educated, did *not* get by economically and relied on society’s help. She was conceived as someone prioritizing her own life over the child’s life.

## Discussion

### Motherhood as a social construction

Research on motherhood often emphasizes biological aspects, such as pregnancy [58], post-natal depression [59], or breastfeeding [60]. The findings revealed little about these biological aspects.

Focusing on biological aspects has been criticized for neglecting interests and power relations that have made women responsible for parenting [61, 62]. For example, a labor market in which women are expected to raise children and men are expected to provide [63]. From this perspective, gendered expectations and characteristics are considered to have been socially or culturally constructed [63]. These expectations and characteristics may vary in relation to the social or cultural context. The differences in responses of Somali-born mothers compared with the nurses and Swedish-born mothers may be explained by perceptions being constructed or shaped, in relation to the cultural and social context of the respondent. Family policies and welfare systems differ between societies and shape the responsibilities of women differently [64–66]. By the same logic, gendered expectations and responsibilities undertaken may transform for an individual as he or she changes social context; for example, through migration [67–69].

### Focus of motherhood: the child, the mother, the community

Various healthcare studies emphasize the community’s pivotal role for Somali refugees [70, 71]. To talk in terms of Somalis as *one* group is of course a simplification. However, it is argued that the indigenous philosophies are deeply embedded in Somali societies – both within Somalia and the Somali diaspora – and in governing Somali peoples’ way of life [72]. Vital in these philosophies is communalism and the individual’s relationship with the community, including social cohesion and collective responsibility [72, 73]. Consequently, social networks and the community are crucial for care if one is ill or in an adverse life situation [74–76]. Consequently, healthcare information provided by friends and key actors in the community may be just as important as information from healthcare staff [77]. This may be why many Somali-born mothers’ responses concerning both everyday and perfect motherhood focused on the surrounding community rather than themselves or their children.

In contrast, the child health nurses generally focused on the mother herself when talking about perfect and everyday motherhood. This is somewhat surprising, given the official position [27, 29] that child health centers emphasize the child’s central position. Of course, the study’s statements explicitly addressing motherhood could have affected the nurses’ responses.

A related deviation among the groups was their expectations (or not) of a mother’s ability to share childcare responsibilities. Despite talking about motherhood within a wider community context, the Somali-born mothers believed that a perfect mother as well as an everyday mother was not supposed to ask others for help when it came to taking care of the child. She was expected to be independent and to manage child-rearing herself. Previous research has suggested that parenting responsibilities are shared to a greater extent between Somali couples living in Finland than is normally the case in Somalia [67]. Studies set in the United States [68] and Sweden [69] argue that the shift toward more equal gender roles between Somali couples in the host country compared to Somalia, may strain relationships; while both partners are expected to work, the husband may be reluctant to share household and parenting responsibilities. The nurses generally responded that mothers *should* cooperate with others in raising children, not least the parents-in-law. The Swedish-born mothers mentioned cooperation, but limited to the partner.

### The meta-mother

Asking the respondents to complete the statement about *perfect* motherhood may be biased as it could be interpreted as being a stereotypical standard of perfection to which their own mothering should be measured. The stereotype of perfection was frequently addressed by Swedish-born mothers as well as nurses in responses such as, ”there’s no such thing as a perfect mother.” But almost every time the impossibility of perfection was mentioned, it was followed by a statement along the lines of “…but she is doing the best she can,” or “…but she always tries her best.” In this sense, the ideal of motherhood is argued not to exist in “real life” but rather as a stereotype yet it impacts the expectations of mothering, as exemplified in the previous quotations that she is always, and in every situation, supposed to do her best given her circumstances. From the nurses’ perspective, the perfect mother also realized when it was time to seek help, or when she could not manage to take care of the child herself. Previous scholars have focused on motherhood as being filled with stress, ambivalence, frustration, and self-blame [78, 79]. Our findings suggest that mothers may be greatly impacted by the concept we termed *meta-mother* – a woman who instinctively knows how and when to act, and who is always giving her maximum in her role as a mother.

### Consequences for the nurse-visitor interaction

The differences and similarities described among the groups in this study may affect the individual meeting between the nurse and the visiting mother at a child health center. A good meeting has been identified as a prerequisite for the child health nurse to find out what the mother desires and to fulfill her expectations [80]. However, the nurses at the Swedish child health centers have been found to initiate most of the topics discussed [81], which may contribute to parents’ lack of healthcare information related to their own concerns [33]. Moreover, it has been pointed out that normative and gendered perceptions risk being transferred to visitors at the child health centers [28]. Official reports focus attention not only on the centers’ visitors, but also on staff and the prevailing norms within the organization [27]. In this study, the nurses both explicitly and implicitly expressed normative perceptions.

The findings of this study suggest that increased knowledge about perceptions of motherhood and engagement with the local communities may help to improve equitable access to healthcare through approaches that are embedded in the local community context. Thus, information about child health services should not be limited to the centers, but disseminated in the wider community [16, 17]. Given the potentially strong role the community can play, those who already have a position of authority or trust in the local community should be used to disseminate information [16, 17, 74].

Of the 35 responding nurses, ten indicated the belief that a partner is part of parenting. A few nurses mentioned the sex of this other being as male, but most mentioned a sexless “partner.” A reason most nurses did not heteronormatively assume the partner to be male may be due to child health service guidelines putting attention on family constellations other than heterosexual families [27]. However, the nurses did not expect the mothers to be *without* a partner. Previous research highlights the fact that single-parent families are increasingly common in Sweden, and investigators have reported resulting disadvantages for the health of the child [82], as well as for single-parenting mothers [83].

Some normative perceptions were explicit. For instance, the nurses expected the mother to ask for help concerning her child. However, Somali-born mothers’ perceptions of ideal motherhood were of someone capable of taking care of the child herself, not asking for help. Moreover, the nurses’ expressions of everyday motherhood were described in rather dashing terms, such as *wonderful* and *beautiful*. Swedish-born mothers responded about perfect motherhood in “positive” terms. However, their responses concerning everyday motherhood were much more nuanced. Somali-born mothers rarely mentioned “positive” characteristics of everyday motherhood. There is a risk that the needs of mothers who express fatigue or being irritated will not be met by nurses at the centers. Given the potential impact of the “meta-mother,” nurses should be aware of the stereotype of the “perfect mother” and therefore be ready to support mothers where they are.

### Limitations of the study

In recent years, Scandinavian scholars have focused attention on fathers’ perceptions, and parental experiences [31, 32]. Still, most appointments to the child health center are made by the mother [31]. The mother is the norm and has been a major influence on the shaping of centers over the years. Understanding the needs and expectations of visitors other than mothers (fathers, for example) requires that one understand the norm. Focusing on motherhood risks reinforcing the mother’s role as the main childcare provider. There is a similar risk of perpetuating stereotypes and generalizations of Somali-born mothers based simply on country of birth. Indeed, these women’s experiences may be very different, depending on the reasons for migration, birth at migration, and other factors. These factors were not considered in this study.

The intent of the current study was to understand perceptions of motherhood in three groups in Western Sweden with participants completing open-ended statements about ideal and everyday motherhood. As such, the scope was limited and therefore caution should be exercised when generalizing the results and conclusions to other populations. Neither should the results be generalized to all Somali-born or Swedish-born women in Sweden.

As with all qualitative studies, there are limitations with smaller sample sizes and nonrandom sampling. In this case, the Somali-born sample was much smaller due to translation resource intensity and logistical constraints of accessing the mothers at a time convenient to them. Data was collected at different sites by necessity in order to access mothers and nurses who were willing to participate in the study.

The responses of the Somali-born mothers were translated into Swedish by an interpreter. The Swedish translations were then translated into English by EE. Consequently, there is a greater risk that information has been lost in translation or somewhat distorted for the Somali-born mothers than for the other two groups (for which translation only took place once).

No demographic information was collected for the nurses. Previous research [84] has suggested that healthcare providers’ backgrounds also affect preconceptions. Previous research [67–69] also indicated that gendered responsibilities transform with migration. In the case of the Somali-born mothers, this has not been thoroughly addressed in this paper, which focuses primarily on differences between groups.

## Conclusion

With the growing emphasis on individualization in healthcare comes a need to acknowledge the social context, including societal structures and norms. This paper suggests that between groups of people – and not solely between single individuals – there are differences and similarities in perceptions of motherhood which potentially may have implications for health services access and utilization. In this study, Somali-born and Swedish-born mothers, as well as nurses expressed differences in the focus of motherhood: the community, the child, and the mother herself. Potential convergences and divergences of beliefs by mothers and staff may constitute a source of misunderstanding, and normative or stereotypical encounters. However, recognition of the existence of gendered and cultural constructions may be a first step to avoiding such encounters. Because healthcare encounters do not take place in a social vacuum, healthcare needs to be provided that is relevant to specific groups of the population and that is grounded within their social context.

Moreover, group perceptions should be used constructively. When healthcare providers design services that satisfy the needs of a diversity of users, equity in healthcare may be enhanced.
